# Experimental and Theoretical Studies of Hydrogen Storage in LaNi_4.4_Al_0.3_Fe_0.3_ Hydride Bed

**DOI:** 10.3390/ma16155425

**Published:** 2023-08-02

**Authors:** Chaker Briki, Dmitry Dunikov, Maha M. Almoneef, Ivan Romanov, Alexey Kazakov, Mohamed Mbarek, Jemni Abdelmajid

**Affiliations:** 1 Laboratory of Studies of Thermal Systems and Energy, Ibn Eljazzar Road, National Engineering School of Monastir, University of Monastir, Monastir 5019, Tunisia; abdelmajidjemni1@gmail.com; 2 Joint Institute for High Temperatures of the Russian Academy of Sciences, Izhorskaya 13, building 2, 125412 Moscow, Russia; ddo@mail.ru (D.D.); kazakoffalex09@gmail.com (A.K.); 3 Department of Physics, College of Science, Princess Nourah Bint Abdulrahman University, Riyadh 11671, Saudi Arabia; mmalmoneef@pnu.edu.sa; 4 Laboratoire de Recherche, Synthèse Asymétrique et Ingénierie Moléculaires des Matériaux Nouveaux Pour L’électroniques Organiques (LR18ES19), Faculté des Sciences de Monastir, Université de Monastir, Monastir 5019, Tunisia; mohamedmbarek99@yahoo.fr

**Keywords:** hydrogen storage, absorption/desorption isotherms, LaNi_4.4_A_l0.3_F_e0.3_ alloy, numerical model, thermodynamic functions

## Abstract

In this article, the experimental measurements of the absorption/desorption P–C–T isotherms of hydrogen in the LaNi_4.4_Fe_0.3_Al_0.3_ alloy at different temperatures and constant hydrogen pressure have been studied using a numerical model. The mathematics equations of this model contain parameters, such as the two terms, n_α_ and n_β,_ representing the numbers of hydrogen atoms per site; N_mα_ and N_mβ_ are the receptor sites’ densities, and the energetic parameters are P_α_ and P_β_. All these parameters are derived by numerically adjusting the experimental data. The profiles of these parameters during the absorption/desorption process are studied as a function of temperature. Thereafter, we examined the evolution of the internal energy versus temperature, which typically ranges between 138 and 181 kJmol^−1^ for the absorption process and between 140 and 179 kJmol^−1^ for the desorption process. The evolution of thermodynamic functions with pressure, for example, entropy, Gibbs free energy (G), and internal energy, are determined from the experimental data of the hydrogen absorption and desorption isotherms of the LaNi_4.4_A_l0.3_F_e0.3_ alloy.

## 1. Introduction

The world’s fossil fuel consumption needs are growing in a huge way, which leads to environmental contamination and climate change. Therefore, we are transitioning from an energy system based on fossil fuels to a renewable energy system [1,2,3,4]. Hydrogen seems to be useful as an energy source and is regarded as an important strategy for transitioning to the efficient use of renewable energy resources [5,6,7]. One of the great difficulties in using hydrogen as an energy source is its storage technique. Even though finding solutions to hydrogen storage attracts the interest of several scientific researchers, various hydrogen storage methods exist, each with its advantages and limitations. Current storage options include compressed gas, liquefied hydrogen, chemical hydrides, and solid-state storage materials. However, each method has its own specific challenges, such as energy efficiency, weight, operating conditions, or material availability. Developing storage technologies that overcome these limitations and offer high efficiency, safety, and practicality is an ongoing area of research [8,9,10,11,12]. Furthermore, the hydrogen is stored in a solid state within the metal hydride bed, providing increased safety at favorable temperatures and pressures. The compounds of the LaNi_5_ type and their derivatives have been examined as useful compounds to store hydrogen [8,9,10,11,12].

Similarly, organic metal compounds have shown promise for hydrogen storage because of their specific properties, including their porous surface and their ability to adsorb hydrogen gas by physisorption [13]. At the same time, these compounds have to be solved in order to become sustainable materials for storing hydrogen.

To overcome these challenges, researchers are exploring several strategies, such as exploring new structures of these types of compounds and experimental techniques of development.

Zhang et al. [14] show that the use of Nb_2_O_5_ nanoparticles grafted on MOF improved the storage behavior of hydrogen in MgH_2_ and lowered the reaction temperatures during the processes of absorption and desorption. Chao Wan et al. [15] are developing an Ag_0.1_Pd_0.9_/N-ompg-C_3_N_4_ nanoparticle as a catalyst for hydrogen storage applications. They prove that this compound exposes a great activity with a rotational frequency value of 1588.2 h^−1^ and activity decreasing after 10 cycles.

The substituted derivatives of the LaNi_5_ compounds are distinguished by their high capacity and their hydrogen atom reversible reaction. As a result, several studies in the literature suggest that nickel partial substitution with other elements (Al, Fe, Mn, and so on) may change several thermodynamic properties of the hydride phases [16,17,18,19,20,21,22]. The partial substitution of these elements for nickel has had significant effects on the absorption and desorption kinetics of hydrogen, cycling performance, and plateau pressure.

The knowledge concerning the metallic hydride’s thermodynamic stability is explained by the isotherms pressure–composition–temperature (P–C–T), which represents the relationship between the equilibrium pressure of hydrogen and the absorbed hydrogen quantity at constant temperatures. Several theoretical models have been proposed by various authors to adequately explain the experimental behavior of hydrogen absorption and desorption by this type of metal compound [23,24,25,26]. The adopted strategy can be made up by fitting the experimental isotherms P–C–T data via mathematical models. Various researchers have worked out to propose several theoretical models to investigate the hydrogen absorption of P–C–T isotherms and desorption by alloys. These models have been fine-tuned to accurately represent the progress of MH in view of the pressure of hydrogen [23,24,25,26,27,28,29,30,31,32].

From the literature, it is observed that a significant amount of research has been carried out on the development of numerical models suitable for experimental studies to fully explain the processes of isotherms of hydrogen sorption by metal hydrides. Furthermore, most researchers are based on the formalism of statistical physics to test several hydrogen storage alloys, which mainly include the LaNi_5_ alloy and its derivatives.

The main objectives of this article are a morphological study of a new LaNi_4.4_Fe_0.3_Al_0.3_ alloy before hydrogenation. Subsequently, we studied the performance of the LaNi_4.4_Fe_0.3_Al_0.3_ sample as a promising hydrogen storage material. In parallel, we proposed an analytical model capable of analyzing the physicochemical characteristics of H absorption–desorption isotherms, referred to as the formalism of the great canonical set of statistical physics. This model determines the number of hydrogen atoms per site and the densities of interstitial sites in the alloy network. This theoretical study provides good agreement with the experimental results.

In addition, the chosen model makes it possible to calculate the thermodynamic functions that govern the absorption–desorption processes of hydrogen. These functions provide information on the energetic and thermodynamic aspects of hydrogen storage, including internal energy, entropy, and Gibbs free energy. By calculating and analyzing these thermodynamic functions, we can improve our understanding of the fundamental principles governing the experimental processes of hydrogen absorption–desorption by the LaNi_4.4_Fe_0.3_Al_0.3_ alloy.

## 2. Materials and Experiment Setup

A 50-gram sample of LaNi_4.4_Fe_0.3_Al_0.3_ alloy was prepared using an arc-melting technique in a furnace under an argon atmosphere. The raw materials, including La, Ni, Fe, and Al, utilized in this experiment have a commercially available purity greater than 99.5% [31]. An ingot was melted three times to obtain high homogeneity. The prepared ingot was manually cleaned from surface oxide layers and ground mechanically. Part of the powder was sent for XRD analysis, and 50 g was selected for PCT measurements. The grinding was performed in an agate mortar to create a powder with a particle size of fewer than 40 microns (controlled by a 40-micron sieve) for XRD investigations. For PCT measurements, particle size was 3–5 mm, and further dispersion proceeded during activation.

The crystal structure and phase composition of the alloy were examined by the D8 Advance (Bruker) diffractometer (Aubrey, TX, USA) with Cu K-alpha radiation. The step size was 0.02°, and the exposition time was one second (1 s). The 2θ angles were reported from 15 to 80°. Rietveld refinement of XRD patterns was conducted by Jana in 2006 using Win PLOTR software (version 3.5d Oct98-LLB-JRC Juan Rodriguez-Carvajal Leon Brillouin (CEA-CNRS)). From the results of XRD, we conclude that the sample is a single-phase alloy with a hexagonal CaCu_5_ type crystal structure. The highest degree of crystallinity can be established by the narrow diffraction peaks of the sample. Refinement of the XRD data shows that the experimental and calculated patterns agree well with each other.

**Figure 1:**
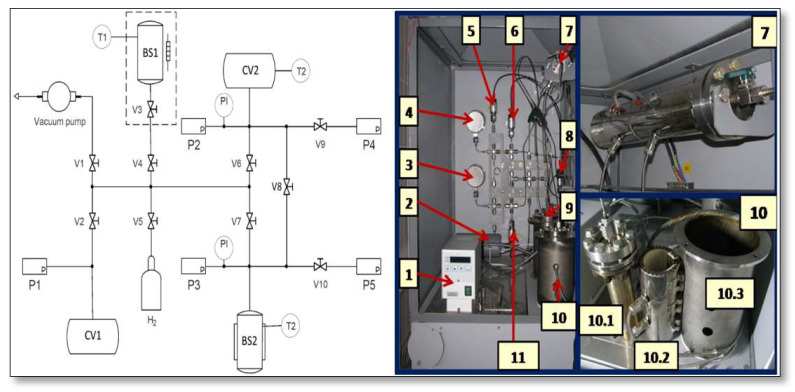
Schematic structure of US150 apparatus for P–C–T measurements. BS1-LaNi_5_ hydrogen accumulator. 1—thermostat (−30–150°); 2—low pressure vessel CV1; 3, 4—manometers; 5—pressure gauge P4; 6—pressure gauge P2; 7—LaNi_5_ hydrogen accumulator BS1; 8—pressure gauge P1; 9—buffer autoclave; 10—autoclave; 10.1—autoclave, 10.2—electric heater, 10.3—casing with thermal isolation; 11—pressure gauge P3.

On a 50 g sample, absorption and desorption isotherms were gauged using a volumetric technique in a Sieverts-type US150 apparatus [32,33], as shown in Figure 1.

The samples were placed into the autoclave for MH samples (CV2 in Figure 1) and evacuated by the Drytel 1025 vacuum pump at an elevated temperature. Hydrogen during measurements is provided by the LaNi_5_ hydrogen accumulator (CV1). The activation procedure of the alloy covers 10 successive H_2_ absorption cycles at pressure P = 5 MPa and a temperature of T = 353 K and desorption for 4 hours for each activation cycle.

Absorption measurement procedure:

- A portion of hydrogen is provided from the LaNi_5_ accumulator BS1 to the buffer vessel CV2.
- The amount of hydrogen in CV2 and auxiliary volumes is calculated by the equation of state for hydrogen developed by Hemmes et al. [34].
- The buffer vessel CV2 is connected to the working autoclave BS2 and some hydrogen is absorbed by the MH sample.
- The system is equilibrated until pressure and temperature remain constant for at least 30 min.
- The amount of hydrogen in CV2, the gas phase of BS1, and auxiliary volumes are calculated, and the difference is attributed to absorbed hydrogen.
- The next portion of hydrogen is provided to CV2 and the procedure is repeated.
- Desorption is measured in a similar manner:
- The sample is charged with a known amount of hydrogen, either in the previous absorption measurement or by a large portion of CV2 at elevated pressure.
- The sample in BS2 is equilibrated.
- Buffer autoclave CV2 is evacuated and then connected to BS2, and a small portion of hydrogen is removed from BS2.
- The system is equilibrated, and the amount of desorbed hydrogen is calculated.
- The procedure is repeated until the end.

**Figure 2:**
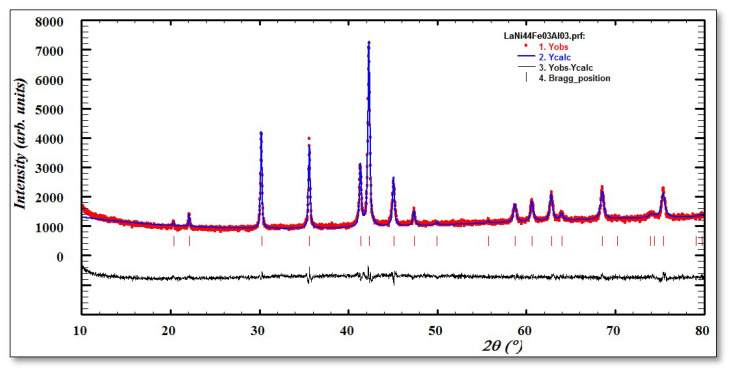
XRD of the LaNi_4.4_Fe_0.3_Al_0.3_ alloy.

Long heat treatment is usually required for multiphase alloys, such as Laves phases, RE-Mg-Ni alloys, and BCC-alloys. After proper melting procedures, LaNi_5_-based alloys are generally crystallized into a single-phase CaCu_5_-type structure. The great homogeneity of the LaNi_4.4_Fe_0.3_Al_0.3_ alloy can be observed from the XRD micrograph (Figure 2), and this alloy has a well-melted single-phase structure of the CaCu_5_ type.

Before the activation process, the metal hydride powders of the LaNi_4.4_Fe_0.3_Al_0.3_ sample bed were subjected to several characterization techniques to analyze their properties. The XRD analysis detects the characteristic X-rays emitted by elements in the sample, providing information about the elemental composition of the LaNi_4.4_Fe_0.3_Al_0.3_ powder (Figure 2). This analysis helps to confirm the presence of the desired elements in the sample.

**Figure 3:**
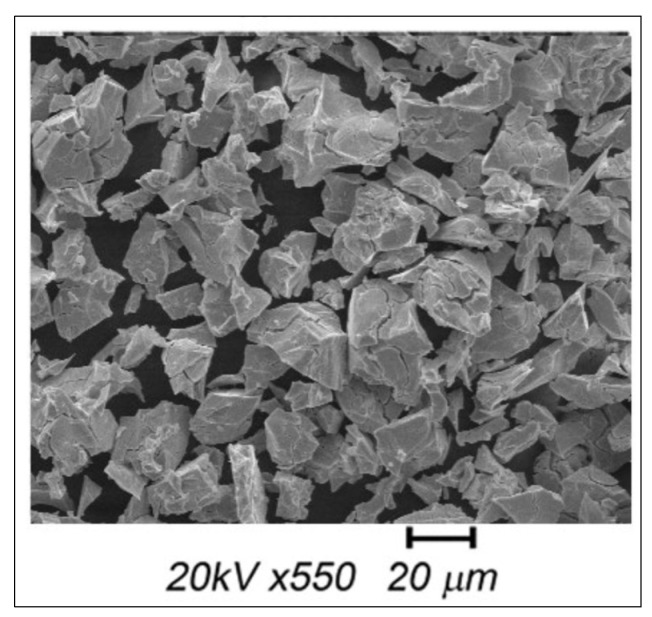
SEM image of the LaNi_4.4_Fe_0.3_Al_0.3_ alloy.

The powders were examined using a scanning electron microscopy (SEM) instrument (Company MEBE FEI Q250 Thermo Fisher Scientific (Waltham, MA, USA) specifically the Tuscan VEGA model. The SEM allows for high-resolution imaging of the sample’s surface morphology. It provides detailed information about the particle size, shape, and surface characteristics of the LaNi_4.4_Fe_0.3_Al_0.3_ powder (Figure 3).

The particle size studies indicated a similar particle size distribution for the alloy. It is also observed in Figure 3 that more than 80% of the particles are arranged in the diameter range of 10 to 30 µm. The harmonic mean diameter of the LaNi_4.4_Fe_0.3_Al_0.3_ alloy is about 12.27 µm.

By employing these characterization techniques, including SEM with XRD, we gained valuable information about the particle size, permeability, and porosity of the LaNi_4.4_Fe_0.3_Al_0.3_ sample bed. These analyses contribute to our understanding of the material’s physical properties.

## 3. Theoretical Modeling

To experimentally study the H absorption–desorption isotherms of the LaNi_4.4_A_l0.3_F_e0.3_ alloy, we used a numerical model based on statistical physics formalism. Based on the work studied by Chaker et al. [23,24,25,26,27,28,29,30] in several articles, we use some approximations in order to simplify the calculations of the equations adapted to the model used. At low pressures that do not exceed 100 bar, we assumed that the hydrogen molecules were ideal gases, and under these conditions, the returned interaction among the gases absorbed or desorbed and the H-H molecules was very slight and could be neglected. Furthermore, each molecule of hydrogen is specified by many freedoms of internal degrees, such as vibratory, rotational, translational, and electronic degrees. Likewise, all the freedom internal degrees applied or undergone by the H_2_ molecule have been ignored, except the translational degree (φ_tr_ = 10–15 K) and rotational degree (φ_rot_ = 85.3 K) only, which are taken into account [26,27,28]. Moreover, the study of the hydrogen absorption–desorption phenomenon needs the involvement of the partition function grand canonical since there is a particle exchange between the free state and the adsorbed one. Therefore, to treat the hydrogen absorption and desorption isotherms (H_2_) by our proposed numerical model, we can keep in view the Na number of H atoms absorbed or desorbed on the Nm, which are detected on the mass unit of the adsorbent.

Absorption and desorption reactions of H_2_ in the interstitial sites of a metal (M) lead to the formation of a hydride according to the following reaction:

(1) $$M+\frac{n}{2}H_{2}\to MH_{n}$$

M indicates the receptor site, n is the number of atoms created per site, MH_n_ represents the formed hydride, and we can see that the element H_n_ is an aggregate of H atoms in the metal alloy.

To explain the phenomenon of H absorption–desorption by the LaNi_4.4_A_l0.3_F_e0.3_ alloy, we can use a numerical model based on the formalism of statistical physics. Furthermore, we have used the grand canonical partition function, Z_gc_, which is determined to identify the interstitial sites of the LaNi_4.4_A_l0.3_F_e0.3_ alloy. This interstitial site can be vacant or occupied by H atoms. The following equation has been used to calculate this grand canonical function [35,36,37,38,39,40,41,42,43,44]:

(2) $$z_{gc}=\sum_{N_{j}}e^{\beta (-\varepsilon_{j}-\mu )N_{j}}$$

where:

- N_j_ represents the occupation state of the receiving site, μ is the chemical potential of the absorbed site, (−ε_j_) represents the receptor site’s desorption energy.
- β is the element determined by the function ($\frac{1}{k_{B}T}$), with k_B_ representing the constant of Boltzmann and T being at 25 °C.

The total Z_gc_ function is dependent on the number of receptor sites per mass unit (N_m_). Therefore, if we assume that these types of sites are independent and equal, then we express the Z_gc_ in the form of a product of two functions:

(3) $$Z_{gc}={\left(z_{gc}\right)}^{N_{m}}={\left(z_{\alpha gc}\right)}^{N_{m\alpha }}\times {\left(z_{\beta gc}\right)}^{N_{m\beta }}$$

$$z_{\alpha gc}=\sum_{Nj=0,1}e^{\frac{-1}{k_{B}T}(\varepsilon_{\alpha }+\mu )N_{j}}=1+e^{\beta (\varepsilon_{\alpha }+\mu )}\;z_{\beta gc}=\sum_{Nj=0,1}e^{\frac{-1}{k_{B}T}(\varepsilon_{\beta }+\mu )N_{j}}=1+e^{\beta (\varepsilon_{\beta }+\mu )}$$

with z_αgc_ and z_ßgc_ being the corresponding partition functions of the two types of sites, which are explicated by the following equations:

The receptor sites occupied by the H atoms density *N_0_* of the *N_m_* is expressed as follows:

(4) $$N_{0}=k_{B}T\frac{\partial \ln(Z_{gc})}{\partial \mu }=N_{m}k_{B}T\frac{\partial \ln(z_{gc})}{\partial \mu }$$

Since the studied system is in thermodynamic equilibrium, the *μ_m_* = *μ*/*n* means chemical potential.

With *n* defined as the number of H atoms per site, and *μ_m_* is assigned to a free H molecule expressed by:

(5) $$\mu_{m}=k_{B}T\ln\left(\frac{N}{z_{g}}\right)$$

where

(6) $$z_{g}=z_{gtr}\times z_{grot}={\left(\frac{2\pi mk_{B}T}{h^{2}}\right)}^{3/2}\times \frac{TV}{2\theta_{rot}}$$

where *θ_rot_* identifies the rotational temperature, m is the mass of an H, *V* is the volume of the gas, and h is nearly 6.626070040 × 10^−34^ J/s.

The translational partition function per unit of volume *z_gtr_* is explained versus vaporization energy Δ*E^v^* and the saturated vapor pressure *P_vs_*:

(7) $$z_{gtr}=V{\left(\frac{2\pi mk_{B}T}{h^{2}}\right)}^{3/2}=\beta P_{vs}e^{\frac{\Delta E^{v}}{RT}}$$

with

(8) $$P_{vs}={\left(\frac{2\pi mk_{B}T}{h^{2}}\right)}^{3/2}\left(k_{B}T\right)e^{\frac{-\Delta E^{v}}{RT}}$$

We can also determine the rotational partition function *z_grot_* by:

(9) $$z_{grot}=\frac{T}{2\theta_{rot}}$$

We can evaluate the occupied sites average numbers via the present equations:

$$N_{0\alpha }=\frac{N_{m\alpha }}{1+{\left(\frac{P_{\alpha }}{P}\right)}^{n_{\alpha }}}\;\mathrm{and}\;N_{0\beta }=\frac{N_{m\beta }}{1+{\left(\frac{P_{\beta }}{P}\right)}^{n\beta }}$$

where

- *n*_α_ and *n*_β_ are the numbers of atoms per site.
- $P_{\alpha }=k_{B}TZ_{g}\;e^{-\beta }{}^{\varepsilon_{m\alpha }}$ and $P_{\beta }=k_{B}TZ_{g}\;e^{-\beta }{}^{\varepsilon_{m\beta }}$ are the pressures at half-saturation, respectively, of the first a and the second type of receptor sites, β.

As well, we can compute the absorbed and desorbed H atoms average number using this equation: $N_{0}=n_{\alpha }N_{0\alpha }+n_{\beta }N_{0\beta }$.

We base ourselves on the equilibrium conditions that correspond to the chemical potential expressed in Equation (5). The filled sites’ average number is represented by this formula:

(10) $$N_{O}=\frac{N_{m\alpha }}{1+e^{{\left(-{\beta \varepsilon }_{\alpha }{}_{m}+\mu_{g}\right)}^{n_{\alpha }}}}+\frac{N_{m\beta }}{1+e^{{\left(-{\beta \varepsilon }_{\beta }{}_{m}+\mu_{g}\right)}^{n_{\beta }}}}=\frac{N_{m\alpha }}{1+{\left(\frac{k_{B}Tz_{g}e^{-\beta \varepsilon_{n_{\alpha }}}}{P}\right)}^{n_{\alpha }}}+\frac{N_{m\beta }}{1+{\left(\frac{k_{B}Tz_{g}e^{-\beta \varepsilon_{n_{\beta }}}}{P}\right)}^{n_{\beta }}}$$

- ε_mα_ and ε_mß_ are two energy functions of an entirely absorbed or desorbed molecule from the two different sites. In fact, these two functions can also be mentioned as follows: ε_αm_ =ε_α_/n_α_ and ε_ßm_ = ε_ß_/n_ß_.

Finally, the global equation of the absorbed or desorbed H atoms average number as a function of pressure can be expressed by:

(11) $$N_{0}=N_{0\alpha }+N_{0\beta }=\frac{N_{m\alpha }}{1+{\left(\frac{P_{\alpha }}{P}\right)}^{n_{\alpha }}}+\frac{N_{m\beta }}{1+{\left(\frac{P_{\beta }}{P}\right)}^{n_{\beta }}}$$

The general formula of the H absorbed and desorbed amount in view of the filled sites’ average number N_0_ is presented by [28]:

(12) $$\left[\frac{H}{M}\right]=n_{\alpha }N_{0\alpha }+n_{\beta }N_{0\beta }$$

After that, the absorbed and desorbed H amount versus the pressure equation is given by this form:

(13) $$\left[\frac{H}{M}\right]\left(P\right)={\left[\frac{H}{M}\right]}_{\alpha }\left(P\right)+{\left[\frac{H}{M}\right]}_{\beta }\left(P\right)=\frac{n_{\alpha }N_{m\alpha }}{1+{\left(\frac{P_{\alpha }}{P}\right)}^{n_{\alpha }}}+\frac{n_{\beta }N_{m\beta }}{1+{\left(\frac{P_{\beta }}{P}\right)}^{n_{\beta }}}$$

(14) $${\left[\frac{H}{M}\right]}_{sat}={\left[\frac{H}{M}\right]}_{\alpha sat}+{\left[\frac{H}{M}\right]}_{\beta sat}$$

Ultimately, the ${\left[\frac{H}{M}\right]}_{sat}$ per unit is mentioned by [45]:

(15) $$\left[\frac{H}{M}\right]\left(P\right)=\frac{{\left[\frac{H}{M}\right]}_{\alpha sat}}{1+{\left(\frac{P_{\alpha }}{P}\right)}^{n_{\alpha }}}+\frac{{\left[\frac{H}{M}\right]}_{\beta sat}}{1+{\left(\frac{P_{\beta }}{P}\right)}^{n_{\beta }}}$$

This numerical equation of this model contains physical–chemical adjusted parameters that are the n_α_, n_β_, N_mα_, N_mβ_, P_α_, and P_β_.

## 4. Results and Discussion

We based our work on models used in the literature to explain the phenomena of hydrogen absorption and desorption by metal hydrides [23,24,25,26,29,30]. Each model is accompanied by an analytical expression that describes the absorption or desorption process. The experimental absorption–desorption isotherms of H by the LaNi_4.4_Al_0.3_Fe_0.3_ alloy at different temperatures are adjusted by different analytical models (Table 1).

We propose mathematical formulas that are convincing for the experimental isotherm curves. A numerical program carries out this analysis. The mathematical discretization of equations in the developed model was based on statistical physics treatments, and the results agree well with the experimental results. The perfect fitting of the result can be considered when the error ratio (R^2^) is of the order of 95% or higher. The fitting of the results was controlled by the determination coefficient (R^2^) that represents a statistical measure and the proportion of the variance for a dependent variable and explained by an independent variable.

According to the results found in Table 1, it is clear that the adjustment constant, “R^2^”, of the model of the monolayer with two energy levels is of the order of 99%. Therefore, according to these values of R^2^, we have chosen the best model adapted to the experimental data.

This coefficient (R^2^) is known as the multiple correlation coefficient, and it was calculated using the equation given below [40].

$$R^{2}=\left[\left(1-\frac{\sum_{i}^{m}{\left(N_{a_{i},\exp}-\bar{N_{a_{i},\exp}}\right)}^{2}-\sum_{i}^{m}{\left(N_{a_{i},\exp}-\bar{N_{a_{i},Model}}\right)}^{2}}{\sum_{i}^{m}{\left(N_{a_{i},\exp}-\bar{N_{a_{i},\exp}}\right)}^{2}}\right)\times \left[\frac{n_{p}-1}{n_{p}-P}\right]\right]$$

where

- $N_{a_{i},\exp}$ presents the average of the *N_a_* values experimentally measured.
- $\bar{N_{a_{i},\exp}}$ is every value of *N_a_* experimentally measured.
- $\bar{N_{a_{i},Model}}$ is each *N_a_* value predicted by the fitted model.
- *n_p_* denotes the number of experiments carried out, and *p* denotes the number of parameters in the adjusted model.

**Figure 4:**
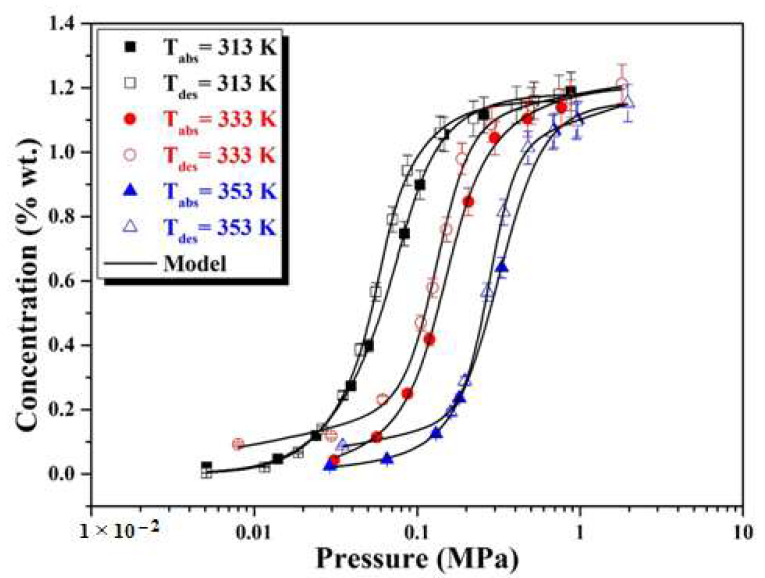
Adjusted absorption and desorption isotherms at different temperatures with the monolayer model with two energy levels.

Figure 4 depicts the fitting curve of the absorption–desorption isotherms of hydrogen by the LaNi_4.4_Al_0.3_Fe_0.3_ alloy at different temperatures using a numerical model.

The R^2^ values are very close to one, indicating that the digital model chosen has great accordance with the experimental values of absorption and desorption isotherms compared with other tested models. Experimental data was fitted to extract the six model parameters: the numbers of H atoms absorbed and desorbed per sites n_α_ and n_β_, the densities of receptor interstitial sites, N_αm_ and N_βm_, and the half-saturation pressures, P_α_ and P_β_.

These parameters can be separated into two categories: the stereographic parameters, n_1_ and n_2_, which describe the anchorage or dis-anchorage of hydrogen atoms to interstitial sites; similarly, the densities N_αm_ and N_βm_, which represent the geometry of receptor sites and stacked atoms in the site, respectively. Parallel to this, the two energetic parameters, P_α_ and P_β_, represent the absorption–desorption energies that define the binding between the hydrogen atoms and interstitial sites.

To deeply understand the characteristics attributed to these parameters, we will examine their evolution versus temperature. Table 2 illustrates the values of these fitted parameters for the absorption–desorption processes, respectively.

### 4.1. Stereographic Parameters

#### 4.1.1. Evolution of Number of Atoms per Site during the Absorption–Desorption Processes

The variation in the number of atoms per site (*n_α_* and *n_β_*) in view of temperature during the hydrogen absorption–desorption is obtained from the two equations (Equations (16) and (17)).

(16) $$\mathrm{Site}\;\alpha :\;\frac{1}{2}n_{\alpha }H_{2}+M_{1}↔n_{\alpha }H+M_{1}↔M_{1}H_{n_{\alpha }}$$

(17) $$\mathrm{Site}\;\beta :\;\frac{1}{2}n_{\beta }H_{2}+M_{2}↔n_{\beta }H+M_{2}↔M_{2}H_{n_{\beta }}$$

The stoichiometric coefficients of the absorption/desorption equation are designed, respectively, n_α_ and n_β_.

**Figure 5:**
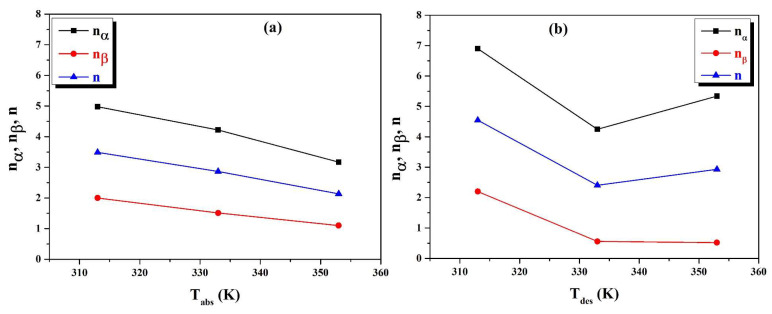
(**a**,**b**): n_α_ and n_β_ comportments as a function of the temperature of in the absorption and desorption.

The two subfigures (Figure 5a,b) show that there are two distinct dynamics in the hydrogen absorption–desorption process, which explains the existence of two types of sites. Furthermore, the numbers of hydrogen atoms in the two types of sites are not of the same order of magnitude and do not behave similarly during the temperature evolution during the hydrogenation and dehydrogenation processes.

These curves show that the values of n_α_ and n_β_ decrease as the temperature rises. This behavior is explained by the difference in the absorption and desorption energies between the two types of sites. Furthermore, we can attribute this phenomenon to a large number of site types. The values of n_α_ range from 5 to 3, while the values of n_β_ range from 2 to 1. The n_α_ to n_β_ ratio is of the order of three. This indicates that the hydrogen uptake capacity and dynamics of the second site are three times lower than those of site 1. As a result, the size of the type 1 insertion site is greater than the size of the type 2 insertion site.

As the temperature rises, the numbers of atoms per site, n_α_ and n_β_, decrease slightly. This can be explained by raison of the absorption reaction is exothermic; thus, the release of heat prevents hydrogen atoms from penetrating.

As a result, the size of the sites shrinks. Furthermore, the hydrogen absorption–desorption processes cause a 15 to 25% increase or decrease in the volume of the network during the insertion of the hydrogen atoms [41].

#### 4.1.2. Evolution of the Densities Receptor Sites, N_mα_ and N_mβ_, during the Absorption–Desorption Processes

**Figure 6:**
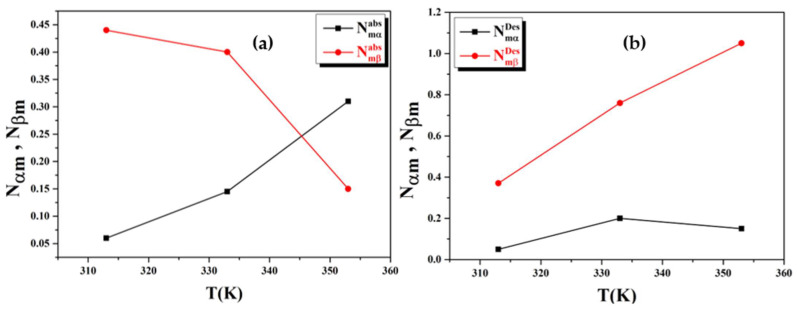
(**a**,**b**): Comportment of the densities receptor sites, N_mα_ and N_mβ_, as functions of temperature during absorption–desorption processes.

In Figure 6a,b, the N_mα_ and N_mβ_ parameters’ evolution in view of temperature during the hydrogen absorption–desorption processes was displayed. These parameters specify the number of sites required to absorb and desorb the required number of hydrogen atoms until saturation is reached.

According to Figure 6a,b, these parameters are in the opposite direction of the n_α_ and n_β_ parameters in view of the T. It is worth noting that the values associated with N_mα_ increase, while the values associated with N_mβ_ decrease through the absorption phenomena and vice versa through the desorption phenomena.

This can be related to thermal agitation, which converts to kinetic energy, as well as the unique nature of hydrogen sorption reactions. Further, in the absorption process, heat is released, which prevents the sites from capturing hydrogen atoms. Correspondingly, the existence of impurities in the gas atmosphere is viewed as just another factor, even though the presence of interstitial site blockage leads to the presence of the impurities on the surface of the compound, all the way through the absorption and desorption processes of H_2_, N_mα_ < N_mβ_ (Figure 6a,b). This explains why the LaNi_4.4_Al_0.3_Fe_0.3_ alloy’s crystalline lattice has more sites in the β phase compared to the α phase. At the same time, the decrepitating phenomenon might be the primary reason for the increase in N_mα_ and N_mβ_ since there are a significant number of sites introduced in the alloy metal following the desorption phenomenon. Such sites are available for hydrogen atom insertion.

#### 4.1.3. Variations of [H/M]_αsat_ and [H/M]_βsat_ during the Absorption–Desorption Processes

**Figure 7:**
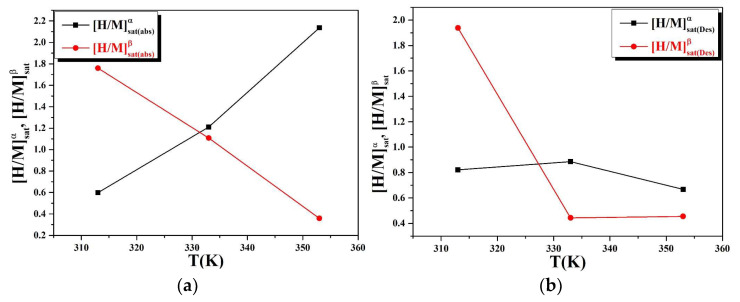
(**a**,**b**): Variations of [H/M]_αsat_ and [H/M]_βsat_ as functions of the temperature of in the absorption and desorption.

The variations of [H/M]_αsat_ and [H/M]_βsat_ with temperature are shown in Figure 7a,b. From this curve, we can see that the amount absorbed and desorbed at saturation in phase α increases with increasing temperature and vice versa for phase β.

The endothermic–exothermic reaction causes the increase and decrease of [H/M]_αsat_ and [H/M]_βsat_ during the absorption–desorption phenomena. Similarly, the insertion of H atoms in the interstitial sites of metal alloys causes a decrepitation of powder grains phenomenon. Given that we used our numerical monolayer model, we can analyze the overall cycle for the absorption and desorption phenomena by using two separate contributions. In this case, we conclude that the characteristics of the two sites differ, but the overall sensation of hydrogen atoms is nearly identical.

### 4.2. Energetic Investigation

The values of energy, Δ*E*^α^ and Δ*E*^β^, are determined from the values of *P*_α_, *P*_β_, and the *P_vs_* of the gaseous hydrogen. The Δ*E*^α^ and Δ*E*^β^ can be explained by:

(18) $$\Delta E^{\alpha }=RT\ln\left(\frac{P_{\alpha }}{P_{vs}}\right)$$

(19) $$\Delta E^{\beta }=RT\ln\left(\frac{P_{\beta }}{P_{vs}}\right)$$

where

- *R*: constant of an ideal gas, which is equal to 8.314472 Jmol^−1^K^−1^;
- *P_vs_*: characterized the vapor pressure of hydrogen gas at saturation; this can be calculated using the following formula:

(20) $$P_{vs}=\exp\left[12.69-\frac{94.896}{T}+1.1125\times \ln\left(T\right)+3.2915\times 10^{-4}\times T^{2}\right]$$

**Figure 8:**
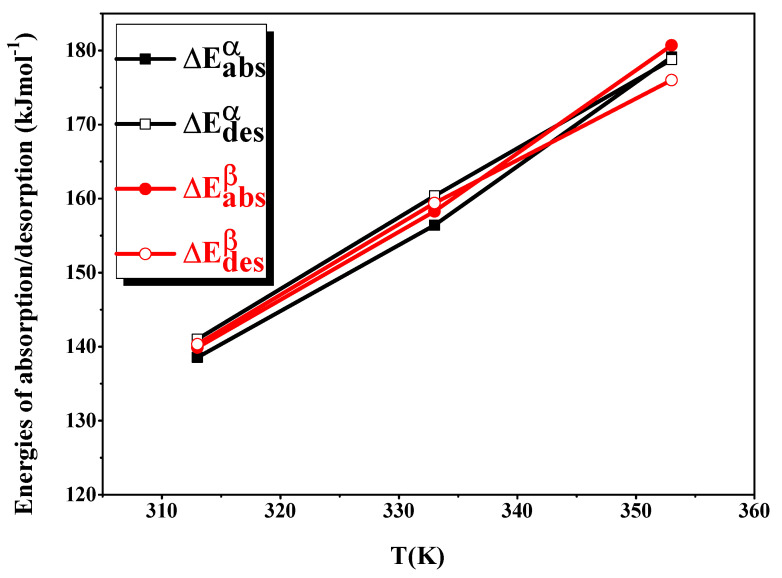
The variation of the absorption–desorption internal energy versus temperature.

Figure 8 depicts the temperature-dependent progression of the absorption–desorption energies of H by the LaNi_4.4_Al_0.3_Fe_0.3_ alloy. In this figure, we can see that the qualities of the absorption and desorption energies (ΔE^α^ and ΔE^β^) increase proportionally to the increase in the temperature.

The absorption–desorption isotherm inflection points are very close to the high-pressure values. As a result, the energies, ΔE^α^ and ΔE^β^, become extremely low, and the pressure plate rises, indicating the importance of high pressures in transferring H from the gas phase to the absorbed state.

### 4.3. Thermodynamic Functions

To fully comprehend the dynamic behavior of the phenomenon of hydrogen absorption and desorption by the LaNi_4.4_Al_0.3_Fe_0.3_ alloy, we must employ a simulation analysis based on statistical physics to investigate the thermodynamic state of the phases α and β.

#### 4.3.1. Internal Energy

The thermodynamic examination of the phenomenon of hydrogen absorption and desorption starts with the behavior of internal energy which includes only the interactions of atoms in the absorption phenomena.

The internal energy is described by the equation [24]:

(21) $$\begin{array}{l} E_{{\mathrm{int}}_{\left(abs/des\right)}}=-\frac{\partial \ln Z_{gc}}{\partial \beta }+\frac{\mu }{\beta }\left(\frac{\partial \ln Z_{gc}}{\partial \mu }\right)\\ =K_{B}T\left(\frac{N_{m\alpha }{\left(\frac{P}{P_{\alpha }}\right)}^{n_{\alpha }}}{1+{\left(\frac{P}{P_{\alpha }}\right)}^{n_{\alpha }}}+\frac{N_{m\beta }{\left(\frac{P}{P_{\beta }}\right)}^{n_{\beta }}}{1+{\left(\frac{P}{P_{\beta }}\right)}^{n_{\beta }}}\right)\left(\ln\left(\frac{\beta P}{Z_{g}}\right)-1\right) \end{array}$$

**Figure 9:**
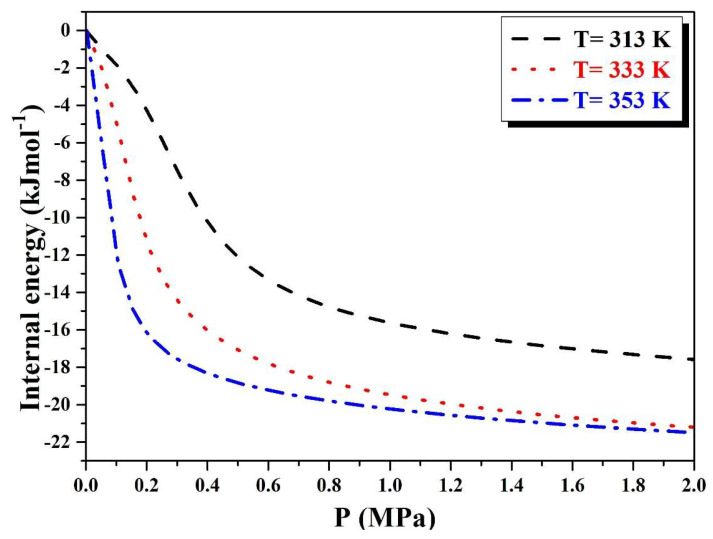
Internal energy evolution with temperature during absorption process.

For the absorption, Figure 9 depicts the variation of internal energy as a function of pressure. We can see that the negative parameters of the absorption’s internal energy, *E*_int_, indicating that the absorption process is more stable than the system’s free state.

Given that ΔE_int_ = E_final_ − E_initial_ is a negative value, this means that when hydrogen atoms are inserted into metal sites, the system produces energy. This energy release is caused by the exothermic nature of the absorption process.

In this case, we say that the metal hydride is interactive because the difference in energy is negative under the system on the condition that the interactions are attractive [42].

#### 4.3.2. The Gibbs Free Energy

The Gibbs free enthalpy G function of H storage by metal hydride LaNi_4.4_Al_0.3_Fe_0.3_ was written using the general expression:

(22) $$G=\mu \left[\frac{H}{M}\right]$$

with *μ* denoting the chemical potential that can be calculated using the following equation:

(23) $$\mu =K_{B}T\ln\left(\frac{\beta P}{Z_{g}}\right)$$

The term *Z_g_* symbolizes the partition function of hydrogen in gaseous form, which is demonstrated by the present expression:

(24) $$Z_{g}=\left(Z_{gtr}+Z_{rot}\right)=\left(\frac{{\left(2\pi mK_{B}T\right)}^{\frac{3}{2}}}{h^{3}}\times \frac{T}{\sigma \theta_{r}}\right)$$

To simplify the equations, we will make the necessary approximations. For that, we will disregard all H_2_ internal degrees in favor of rotation and translation degrees.

The Gibbs free energy is expressed as follows:

(25) $$G=K_{B}T\ln\frac{\beta P}{Z_{g}}\left(\frac{n_{\alpha }N_{\alpha m}}{1+{\left(\frac{P_{\alpha }}{P}\right)}^{n_{\alpha }}}+\frac{n_{\beta }N_{\beta m}}{1+{\left(\frac{P_{\beta }}{P}\right)}^{n_{\beta }}}\right)$$

**Figure 10:**
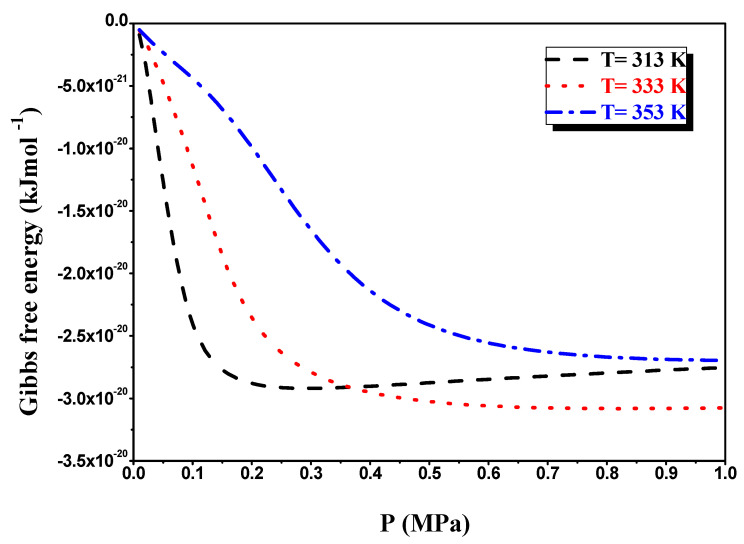
Evolution of the free enthalpy G with pressure during the absorption process.

We discovered in Figure 10 that the Gibbs free energy is always negative regardless of temperature evolution, which explains the nature of the spontaneous hydrogen absorption reaction by the LaNi_4.4_Al_0.3_Fe_0.3_ alloy. Because the hydrogen absorption process is characterized by the release of heat, the system evolves into lower energy and a more thermodynamically stable state [43,44]. Likewise, for the temperatures 333 K and 313 K, the Gibbs energy values overlapped each other at about 0.36 MPa pressure because of the values of the investigated atom hydrogen number in the two phases (α and β), which are not integer values.

#### 4.3.3. Configurationally Absorption Entropy

The absorption entropy function of our studied system is determined using the grand potential expressions, *J* and the *Z_g_* [45,46].

The equation characterizes the *J*:

(26) $$J=-K_{B}T\ln\left(Z_{gc}\right)=E-\mu N-TS$$

where

(27) $$E-\mu N=-\frac{\partial }{\partial \beta }\left(\ln Z_{gc}\right)$$

Combining Equations (26) and (27) yields the following grand potential *J* relationship:

(28) $$J=-\frac{\partial }{\partial \beta }\left(\ln Z_{gc}\right)-TS$$

The entropy *S* is defined by:

(29) $$TS=-\frac{\partial }{\partial \beta }\left(\ln Z_{gc}\right)+K_{B}T\ln Z_{gc}$$

(30) $$S=-\beta K_{B}\frac{\partial }{\partial \beta }\left(\ln Z_{gc}\right)+K_{B}\ln Z_{gc}$$

Finally, the entropy *S* is written as follows:

(31) $$S=S_{\alpha }+S_{\beta }\;S=K_{B}\times \left[N_{\alpha m}\ln\left(1+{\left(\frac{P}{P_{\alpha }}\right)}^{n_{\alpha }}\right)+N_{\beta m}\ln\left(1+{\left(\frac{P}{P_{\beta }}\right)}^{n_{\beta }}\right)-\left(\frac{N_{\alpha m}{\left(\frac{P}{P_{\alpha }}\right)}^{n_{\alpha }}\ln{\left(\frac{P}{P_{\alpha }}\right)}^{n_{\alpha }}}{\left(1+{\left(\frac{P}{P_{\alpha }}\right)}^{n_{\alpha }}\right)}+\frac{N_{\beta m}{\left(\frac{P}{P_{\beta }}\right)}^{n_{\beta }}\ln{\left(\frac{P}{P_{\beta }}\right)}^{n_{\beta }}}{\left(1+{\left(\frac{P}{P_{\beta }}\right)}^{n_{\beta }}\right)}\right)\right]$$

**Figure 11:**
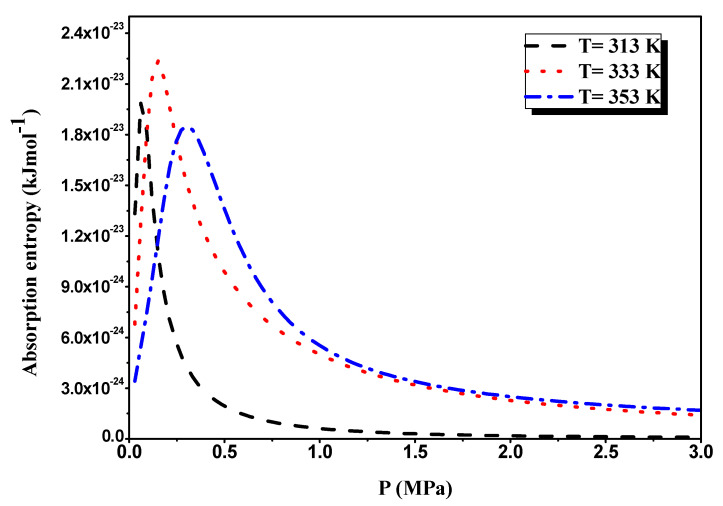
Evolution of the entropy with pressure during the absorption process.

The absorption entropy evolution in view of pressure is depicted in Figure 11. We can see from this graph that absorption entropy variation has two bands, which correspond to each of the two sites of this alloy.

It is clear that the shapes of the two peaks, *P*_α_ and *P*_β_, are clearly accompanied by two distinct comportments before and after the half-saturation pressure. Indeed, for *P* = *P*_α_ = *P*_β_, the evolution of absorption entropy begins at the start with low values of the absorption process and then lands at a maximum value near the half-saturation.

This means that the system’s disorder is nearly at its peak when the hydrogen atoms occupy all the alloy’s interstitial sites. Then, the evolution of entropy diminishes towards low values, and after the values for *P* = *P*_α_ = *P*_β_, the system expands such that the entropy decreases to zero.

## 5. Conclusions

Experimental measurements of hydrogen isotherms in the LaNi_4.4_Al_0.3_Fe_0.3_ alloy were studied using a numerical model based on the formalism of statistical physics. The mathematical equations of this model incorporate various parameters to describe the hydrogen storage behavior.

The profiles of these parameters during the absorption–desorption processes are studied as a function of temperature. This analysis provides insights into how the hydrogen storage behavior evolves within the LaNi_4.4_Al_0.3_Fe_0.3_ alloy at different temperatures.

Furthermore, the evolution of thermodynamic functions with pressure is extracted based on the experimental data of the hydrogen absorption and desorption isotherms. These thermodynamic functions provide valuable information about the energetic and stability changes associated with hydrogen storage in the LaNi_4.4_Al_0.3_Fe_0.3_ alloy.

## Figures and Tables

**Table 1:** R^2^ values for the adjustment of absorption–desorption isotherms of hydrogen in LaNi_4.4_A_l0.3_F_e0.3_.

	Model (R^2^)	Model 1 (Hill)	Model 2 (Monolayer with Two Energy Levels)	Model 3 (Monolayer with Three Energy Levels)
T(K)				
Absorption				
313		0.99213	0.99887	0.99617
333		0.99214	0.99819	0.99715
353		0.99313	0.99941	0.99962
Desorption				
313		0.98625	0.99918	0.98957
333		0.98869	0.99631	0.98776
353		0.98957	0.99967	0.99892

**Table 2:** The adjusted values of the absorption–desorption parameters.

T (K)	n_α_	n_β_	P_α_	P_β_	N_mα_	N_mβ_
Absorption						
313	4.98	2	0.08	0.06	0.06	0.44
333	4.22	1.51	0.148	0.15	0.145	0.4
353	3.17	1.1	0.31	0.18	0.31	0.15
Desorption						
313	6.9	2.2	0.06	0.05	0.05	0.37
333	4.25	0.56	0.13	0.1	0.2	0.76
353	5.34	0.52	0.27	0.9	0.15	1.05

## Data Availability

Exclude this statement if the study did not report any data.
